# Tip-enhanced Raman spectroscopy with amplitude-controlled tapping-mode AFM

**DOI:** 10.1038/s41598-022-17170-7

**Published:** 2022-07-27

**Authors:** Takayuki Umakoshi, Koji Kawashima, Toki Moriyama, Ryo Kato, Prabhat Verma

**Affiliations:** 1 Department of Applied Physics, Osaka University, Suita, Osaka 565-0871 Japan; 2 Institute for Advanced Co-creation Studies, Osaka University, Suita, Osaka 565-0871 Japan; 3 PRESTO, Japan Science and Technology Agency, Kawaguchi, Saitama 332-0012 Japan; 4 Institute of Post-LED Photonics, Tokushima University, Tokushima, Tokushima 770-8506 Japan

**Keywords:** Nanophotonics and plasmonics, Raman spectroscopy, Characterization and analytical techniques

## Abstract

Tip-enhanced Raman spectroscopy (TERS) is a powerful tool for analyzing chemical compositions at the nanoscale owing to near-field light localized at a metallic tip. In TERS, atomic force microscopy (AFM) is commonly used for tip position control. AFM is often controlled under the contact mode for TERS, whereas the tapping mode, which is another major operation mode, has not often been employed despite several advantages, such as low sample damage. One of the reasons is the low TERS signal intensity because the tip is mostly away from the sample during the tapping motion. In this study, we quantitatively investigated the effect of the tapping amplitude on the TERS signal. We numerically evaluated the dependence of the TERS signal on tapping amplitude. We found that the tapping amplitude had a significant effect on the TERS signal, and an acceptable level of TERS signal was obtained by reducing the amplitude to a few nanometers. We further demonstrated amplitude-controlled tapping-mode TERS measurement. We observed a strong dependence of the TERS intensity on the tapping amplitude, which is in agreement with our numerical calculations. This practical but essential study encourages the use of the tapping mode for further advancing TERS and related optical techniques.

Tip-enhanced Raman spectroscopy (TERS) has played a crucial role in nanophotonics and related fields over the past few decades as a great tool for chemical and molecular analysis of samples with a nanoscale spatial resolution by exploiting the light field confined within a nanometric volume at a metallic tip for Raman measurement^1–4^. Locating the metallic tip to a region of interest, one can obtain a Raman spectrum from a nanometric spot to analyze the chemical composition of the samples with nanoscale details. Moreover, super-resolution near-field Raman imaging is possible with a typical spatial resolution of ~ 10 nm by precise raster-scanning of either the tip or the sample^5–8^. It is even possible to achieve sub-nanometer spatial resolutions under specific conditions^9,10^. Therefore, in TERS measurements, position control of the metallic tip with nanoscale precision is essential for stable and reliable measurements, in which atomic force microscopy (AFM) is often employed as one of the most common methods for precise tip position control^1,2,6–8,11,12^. It has several advantages over other scanning probe microscopes that may also be used in TERS. One advantage of this method is its versatility. As it regulates the tip position by sensing van der Waals interactions between samples and a tip, any type of sample, such as an insulator or semiconductor, is measurable, which is not the case for scanning tunneling microscopy that requires conductivity to samples^13^.

Although several operation modes are available for tip control in AFM, the contact mode is often utilized for TERS measurements^1,2,6–8,12,14–18^. This is because the localized near-field light at the apex of the metallic tip is always in contact with the samples, and thus it maximizes the TERS signal intensity by keeping the samples continuously illuminated with the near-field light during measurement. However, one of the drawbacks is that the samples can be scratched or even damaged by tip scanning, especially for soft and fragile samples, such as biomolecules. Another major operation mode in AFM is the tapping mode, that is, amplitude modulation AFM. In the tapping mode, the cantilever tip oscillates vertically with a typical amplitude of a few tens to hundreds of nanometers. The tapping amplitude is reduced when the tip comes on the sample with some height. By monitoring and regulating changes in the tapping amplitude, tapping-mode AFM can precisely control the position and force between the tip and the sample. As the tip gently taps the samples in the tapping mode, damage to the samples is significantly suppressed compared with the contact mode. Therefore, the tapping mode is more common for normal AFM measurements. A recent report suggested that such less perturbation to the samples is crucial for reliable TERS measurements^19^. However, it brings a serious concern for TERS, as it results in low TERS signal intensity because the tip is mostly away from the sample. Near-field light at the tip apex illuminates the samples for an extremely short duration only when the tip comes in the close vicinity of the sample^11^. This is particularly significant when the tapping amplitude is large. In other words, it is possible to improve TERS signal intensity by reducing the tapping amplitude. Tapping-mode AFM has been utilized in TERS, for example, to precisely regulate the tip-sample distance by employing time-gate techniques^20–22^. However, there have not been sufficient studies on elucidating the effect of tapping amplitude on TERS signal intensity^23,24^, which is highly important for reliable TERS measurements to simultaneously achieve low sample damage and acceptable level of signal intensity in the tapping-mode operation.

In this study, we investigated the effect of tapping amplitude on TERS signal intensity and other optical properties in TERS measurements. We first mathematically evaluated the dependence of TERS signal intensity on the tapping amplitude, where we showed the significance of the tapping amplitude and the importance of tapping amplitude control through quantitative numerical analysis. We found that a reasonably high TERS signal intensity compared with the contact mode was obtained by reducing the tapping amplitude to a few nanometers. We conducted TERS measurements by varying the tapping amplitude to experimentally verify the tapping-amplitude dependence. TERS signal intensity increased significantly as the tapping amplitude was reduced. These results provide practical and meaningful insights into the effective application of tapping-mode AFM for TERS measurements.

## Results

**Figure 1:**
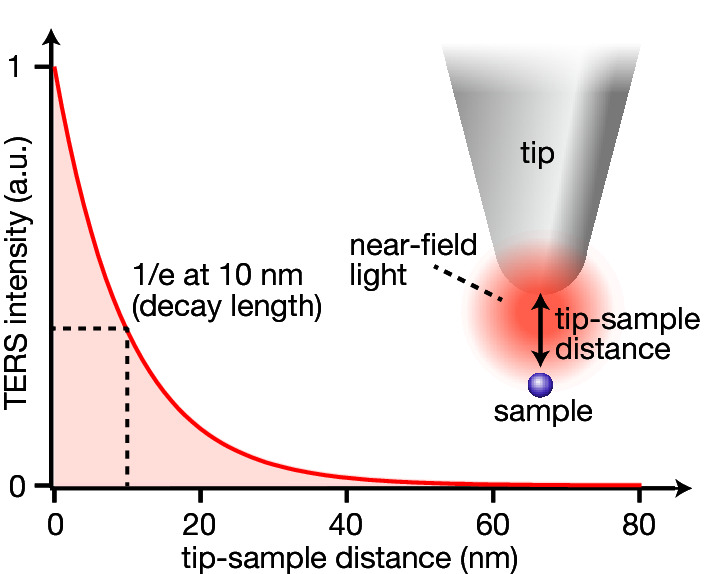
TERS intensity dependence on the tip-sample distance.

Near-field light is strongly localized at the apex of a metallic tip, and thus the near-field light intensity at the sample and the resulting TERS intensity exponentially decreases with the distance between the tip and sample^21^, as shown in Fig. 1. As a function of the tip-sample distance *x*, the TERS intensity *I*_TERS_ is expressed as follows:

$$ I_{{{\text{TERS}}}} = \, I_{0} exp\left( { - \frac{x}{d}} \right) $$

**Figure 2:**
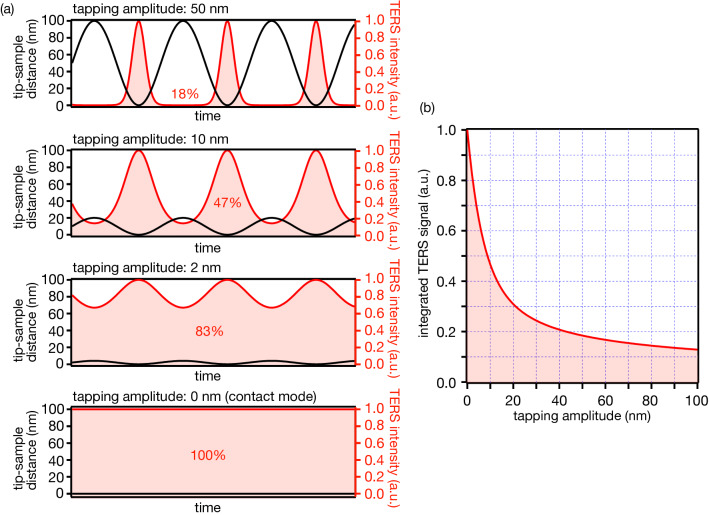
(**a**) Time variation of the tip-sample distance and TERS intensity at different tapping amplitudes. (**b**) Relationship between integrated TERS signal and the tapping amplitude. The TERS signal is normalized by the signal obtained at the 0 nm amplitude, that is, the contact mode.

Here, *I*_*0*_ represents TERS intensity at a tip-sample distance of 0 nm, and *d* indicates the decay length of TERS intensity. To understand the relationship between the tapping amplitude and TERS signal intensity, we chose a fixed value of the decay length as *d* = 10 nm, which is a typical value used in TERS measurements. We evaluated how TERS intensity varied with time under the tapping motion of the tip. Assuming that the tip sinusoidally oscillates with the tapping amplitude *A*, TERS intensity *I*_TERS_ is described as

$$ I_{{{\text{TERS}}}} = \, I_{0} exp\left[ { - \frac{{\left\{ {A + A\sin \left( {\omega t} \right)} \right\}}}{d}} \right] $$

where *ω* and *t* are the tapping frequency and time, respectively. Black and red curves in Fig. 2a represent the time variation of the tip-sample distance and TERS intensity *I*_TERS_, respectively, at different tapping amplitudes. When the tapping amplitude was set to 50 nm, which is a typical value of amplitude used in tapping-mode AFM, the TERS signal was generated with reasonable intensity only for a short duration when the tip was close to the sample. We then decreased the tapping amplitude to 10, 2, and 0 nm, respectively, and compared TERS signals. Here, an amplitude of 0 nm indicates that the tip is operated in the contact mode. By reducing the tapping amplitude from 50 to 0 nm, the total amount of TERS signal drastically increased. The largest TERS signal was obtained in the contact mode, as expected, because the tip-sample distance was always 0 nm, and hence the sample was always immersed in the near-field light. We integrated TERS intensity with time to evaluate the mean TERS signal intensity. As indicated in Fig. 2a, the integrated TERS signal was reduced to 83% at a tapping amplitude of 2 nm compared with the contact mode. It was almost half at an amplitude of 10 nm and dropped down to only 18% at an amplitude of 50 nm. These results show the importance of optimizing the tapping amplitude for TERS measurements. It requires at least five times longer exposure time to obtain a similar amount of TERS signal in the case of a tapping amplitude of 50 nm compared to the contact mode. This is significant especially when attempting to perform TERS imaging, as it takes a long time to acquire multiple TERS spectra. In contrast, we found that a reasonably high TERS signal could be obtained by reducing the amplitude to a few nanometers. Figure 2b shows a relationship between the integrated TERS signal and the tapping amplitude. Here, the integrated TERS signal was normalized to the intensity obtained in the contact mode. This shows how quickly the TERS signal decreases with the tapping amplitude and how crucial it is to maintain the tapping amplitude small. In other words, by reducing the amplitude to a few nanometers, it is possible to obtain affordable TERS intensity or even TERS intensity almost comparable to that obtained in the contact mode, which is a promising fact to apply the tapping-mode AFM for TERS measurements.

**Figure 3:**
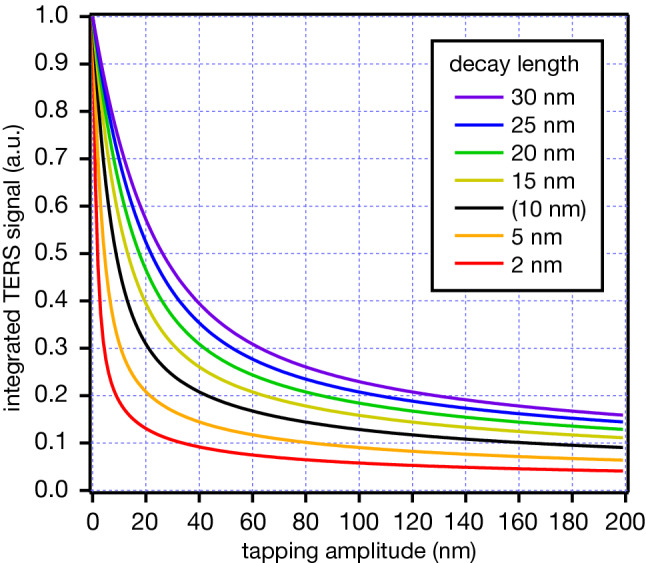
Relationship between integrated TERS signal and the tapping amplitude at different decay lengths of TERS intensity.

In the analysis above, although we assumed that the decay length *d* has a typical value of 10 nm, the decay length should vary between tips because it changes even with a slight difference in the tip shape. Hence, we also investigated the relationship between the TERS signal and the tapping amplitude with different decay lengths. We varied it from 2 to 30 nm, as shown in Fig. 3. The case of a 10 nm decay length is also included for a comparison in Fig. 3, as shown by the black curve. As expected, when the near-field light was strongly confined, that is, when the decay length was short, the influence of the tapping amplitude on TERS signal was significant. In particular, in the case where the decay length was 2 nm, TERS signal was reduced by more than 80% even at a tapping amplitude of 10 nm in comparison to the contact mode. On the other hand, the change in the TERS signal was comparatively moderate with larger decay lengths. When the decay length was 30 nm, the reduction of the TERS signal was less than only 25% at a tapping amplitude of 10 nm, although such a long decay length is rarely obtained with normal metallic tips. However, even if the signal reduction is modest, it is still better to maintain a smaller tapping amplitude for a larger TERS signal. Please note that the results do not imply that a larger decay length provides a higher TERS signal, as it is normalized by the intensity obtained in the contact mode for each decay length. A stronger confinement, *i.e.* a shorter decay length, of near-field light leads to a higher TERS intensity. The results shown in Fig. 3 can be used to compare different tapping amplitudes for the same tip and decay length. However, it is not appropriate to compare different tips that show different decay lengths because the near-field light intensity is completely different between different metallic tips.

**Figure 4:**
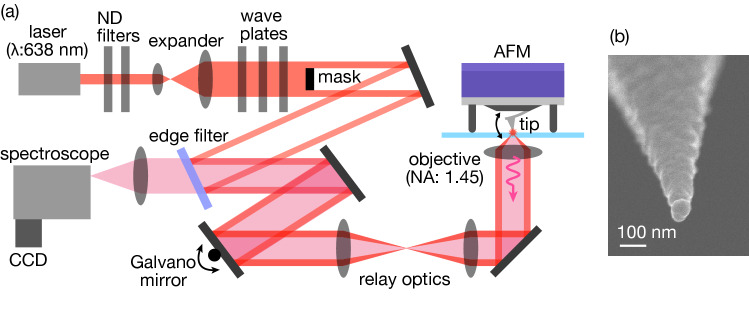
(**a**) Schematic of experimental setup for the tapping-mode TERS measurement. (**b**) SEM image of a metallic tip.

We then performed tapping-mode TERS measurements by changing the tapping amplitude to examine the dependence of the TERS signal on the tapping amplitude. Figure 4a shows a schematic of the experimental setup. A single-mode laser (wavelength: 638 nm) was passed through the beam expander and several filters. A spatial mask was also inserted for evanescent illumination to a metallic tip. The metallic tip was mounted on an AFM, and the laser was focused on the tip through an oil-immersion objective lens (NA: 1.45). The position of the laser focus was precisely adjusted to the tip apex using a Galvano mirror scanner to efficiently excite near-field light at the tip apex. The Raman signal excited by the near-field light was detected using a Peltier-cooled CCD camera through a spectroscope. Rayleigh-scattered noise was efficiently removed using an edge filter. More details about the experimental setup are described in the Methods section and in our previous reports^7,8,11^. Figure 4b shows a scanning electron microscopy (SEM) image of a typical metallic tip. It was fabricated by depositing silver on a commercially available tapping-mode cantilever tip via physical vapor deposition. Granular silver nanoparticles were deposited on the tip, which is suitable for generating strong near-field light through resonance oscillations of localized surface plasmons at the tip apex. The cantilever length was 160 µm (OMCL-AC160TN; Olympus), and the spring constant and resonance frequency are 26 N/m and 300 kHz, respectively.

**Figure 5:**
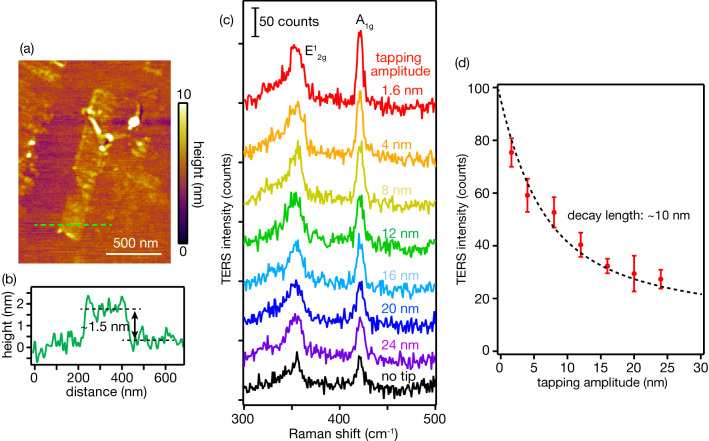
(**a**) AFM image of a bilayer WS_2_ (**b**) Topographic line-profile obtained from the green dotted line in (a). (**c**) TERS spectra of WS_2_ obtained at different tapping amplitudes. The black curve shows the far-field Raman spectrum obtained without a tip. (**d**) Dependence of TERS intensity of A_1g_ mode on the tapping amplitude.

To conduct TERS measurements, we chose tungsten disulfide (WS_2_) as the sample. WS_2_ is an atomically thin two-dimensional material that is promising for future electric devices owing to its thinness and superior electric properties^25–28^. First, we deposited a buffer layer of copper and subsequently deposited a smooth gold layer on a cleaned glass substrate via physical vapor deposition. The thickness of the copper layer was 2 nm^29^. It works as an adhesion layer to smoothen the gold layer. The thickness of the gold layer was 10 nm. Few-layered WS_2_ was mechanically exfoliated from a WS_2_ bulk crystal using scotch-tape and attached to the gold layer^30,31^. Figure 5a shows an AFM image of a bilayer WS_2_ sample used for TERS measurement. The number of layers was determined from its height, as shown in Fig. 5b. The sample was then placed on the AFM sample stage, and the metallic tip was brought on the sample from the top, so that WS_2_ was sandwiched between the gold layer and the metallic tip. TERS measurements were performed in the gap-mode regime, which allows reliable TERS measurements because one can easily obtain a strong enhancement of the near-field Raman signal from the tip^11,12,32^. Although Raman signal must pass through the gold layer in this configuration, the 10-nm-thick gold layer is thin enough for Raman measurement, as confirmed in our previous study^11^.

For the tapping-mode TERS measurements, the tapping amplitude was controlled by changing the applied voltage to an oscillation piezo, and was monitored in the AFM system. The tapping amplitude was varied from 1.6 to 24 nm. Figure 5c shows TERS spectra of WS_2_ obtained at different tapping amplitudes. The laser power at the sample plane and the exposure time were ~ 1.0 mW and 5 s, respectively. WS_2_ exhibited two distinctive Raman peaks originating from the E^1^_2g_ mode at approximately 350 cm^−1^ and the A_1g_ mode at approximately 420 cm^−1^^28,33^. A far-field Raman spectrum acquired by retracting the tip is also shown by the black spectrum. We confirmed Raman signal enhancement by near-field light at the tip apex. More importantly, signal enhancement increased with decreasing tapping amplitude. TERS signal was slightly enhanced or showed almost no enhancement at a tapping amplitude of 24 nm. In contrast, the TERS signal was significantly enhanced at a tapping amplitude of 1.6 nm. The A_1g_ mode was enhanced to three times as high as that of the far-field Raman spectrum. We experimentally verified that the tapping amplitude significantly affected the TERS intensity. TERS signal intensities of the A_1g_ mode are plotted with respect to the tapping amplitude in Fig. 5d. The intensities are plotted after subtracting the far-field Raman spectrum from corresponding TERS spectrum such that Raman signal generated only by the near-field light was extracted. Five TERS spectra were acquired at different locations within the sample for each tapping amplitude. Please note that TERS intensities were almost the same at different locations as our WS_2_ sample had homogeneous structures. It was evaluated that TERS signal was increased three times by reducing the tapping amplitude from 24 nm to 1.6 nm. Such a large improvement allows to obtain a TERS spectrum with high signal-to-noise ratio. As aforementioned, it is also crucial for TERS imaging because it takes a long time to obtain a large number of TERS spectra pixel by pixel. The imaging time can be shortened by simply reducing the tapping amplitude.

## Discussions

**Figure 6:**
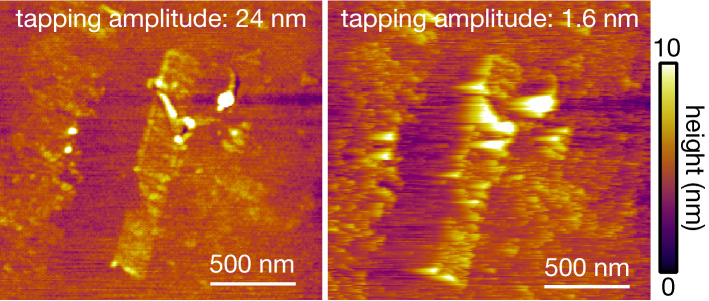
AFM images of WS_2_ acquired at tapping amplitudes of 24 and 1.6 nm.

We confirmed the importance of optimizing the tapping amplitude for higher TERS signal intensities. This suggested oscillating a cantilever with an amplitude of at least a few nanometers or even less. However, in the investigations above, we considered TERS intensity only. In fact, small tapping amplitudes could technically raise some concerns regarding the AFM operation. We discuss some possible issues with small tapping amplitudes. One of the issues that we suspect is that the AFM operation becomes unstable for large samples with a small tapping amplitude. If the sample height is a few tens of nanometers but the tapping amplitude is just a few nanometers, the tip cannot easily pass over the sample. In general, it is suggested that the tapping amplitude should be greater than the sample height. This is one of the reasons why a large tapping amplitude of a few tens of nanometers is commonly used in ordinary AFM measurements in the tapping mode. The height of our WS_2_ sample was as low as ~ 1.5 nm, which is comparable to the smallest tapping amplitude of 1.6 nm in our experiments, but we still observed an issue with the smooth operation of AFM imaging process. Figure 6 shows AFM images of bilayer WS_2_ obtained with tapping amplitudes of 24 nm and 1.6 nm, which were measured with the scan rate of 0.5 Hz/line. The other measurement parameters were also the same in both cases. The figure shows nanoscale details of the WS_2_ sample in the case of 24 nm, whereas the image quality became much worse in the case of 1.6 nm. This is particularly obvious for some areas with larger heights in the image. The small tapping amplitude may cause not only low image quality but also sample damage, as a too small tapping amplitude is almost equivalent to that of the contact mode operation. Therefore, it is crucial to carefully adjust the tapping amplitude to balance a stable AFM operation and TERS signal intensity by considering the physical structures of the samples. Please note that as mentioned above, measurement parameters other than the tapping amplitude are the same for both cases for a valid comparison. The quality of the AFM image obtained with a tapping amplitude of 1.6 nm can be improved by fine tuning other parameters, such as the scan rate, PID feedback parameters and so on.

Even if the samples are low in height and robust, a small tapping amplitude can cause another issue. In tapping-mode AFM, the tapping motion is monitored by a two- or four-segmented photodiode in an optical lever system, in which a diode laser is reflected at the back side of the cantilever and goes to the segmented photodiode. The segmented photodiode was used to monitor the position where the reflected laser hits the photodiode. Because the cantilever is deflected by the tapping motion, the position of the reflected laser changes sinusoidally on the segmented photodiode by following the tapping motion. The cantilever tapping amplitude is evaluated form the sinusoidal position change. When the tip comes on the sample with a certain height, the tapping amplitude is reduced. The tip is then lifted up by the same height as the sample to maintain the tapping amplitude at a certain value through PID feedback. However, if the tapping amplitude is too small, the position of the reflected laser on the photodiode changes by a very small amount, and thus cantilever deflection cannot be properly detected, which makes the AFM operation unstable. Therefore, the applied voltage for oscillating the cantilever was set to a minimum value of 10 mV in our AFM system, at which we obtained a tapping amplitude of 1.6 nm as demonstrated. The tapping amplitude could not be reduced further in our current system.

One possible way to further reduce the amplitude while maintaining a stable AFM operation is to use a shorter cantilever. A shorter cantilever provides a larger deflection of the cantilever compared with a longer cantilever at the same tapping amplitude. Although we used 160-µm-long silicon cantilevers in this study, we attempted several types of cantilevers. For the 160-µm-long cantilever, the cantilever width and thickness were 40 µm and 3.7 µm, respectively, resulting in a spring constant and resonance frequency of 26 N/m and 300 kHz, respectively. As a comparison, when we used a 240-µm-long cantilever silicon tip (OMCL-AC240TN, Olympus) (cantilever width: 40 µm, cantilever thickness: 2.3 µm, spring constant: 2 N/m, resonance frequency: 70 kHz), the minimum tapping amplitude was ~ 20 nm at an applied voltage of 10 mV because it was much longer than the 160-µm-long cantilever. Therefore, it was not possible to observe any signal enhancement and perform detailed studies on the tapping amplitude. We also attempted to use a 55-µm-long cantilever silicon tip, and expected to achieve a tapping amplitude smaller than 1.6 nm (OMCL-AC55TN, Olympus) (cantilever width: 31 µm, cantilever thickness: 2.35 µm, spring constant: 85 N/m, resonance frequency: 1600 kHz). In this case, however, the cantilever was too stiff to oscillate with the oscillation piezo in our system, owing to its shortness and the resulting high spring constant. Therefore, we conclude that the 160-µm-long cantilever provides a good balance between the minimum tapping amplitude and stable AFM operation. Please note that these cantilevers were compared after the silver deposition with the thickness of 60 nm. To minimize influence of the silver deposition on the physical properties of cantilevers, a mask was set during the deposition so that silver was deposited only on the tip at the very end of cantilevers. Although many different types of cantilevers are still commercially available, we intend to leave them for future work. In principle, we expect that a short and soft cantilever will show good performance for tapping-mode TERS measurements with small tapping amplitudes and larger cantilever deflections. Although we anticipated that much smaller tapping amplitude may crease issues with AFM images, as discussed earlier, one can try to find a balancing optimization.

At last, we discuss advantages of the tapping-mode AFM for other optical measurements. In this study, we investigated the effect of the tapping amplitude only on TERS measurements. However, the tapping-mode AFM with small amplitude would be more beneficial for tip-enhanced measurements of photoluminescence or fluorescence. For tip-enhanced photoluminescence/fluorescence measurements, the signal intensity was determined not only by plasmonic signal enhancement but also by signal quenching due to nonradiative energy transfer from samples to metal. The effect of signal quenching is strong when a metallic tip is in physical contact to samples. Although there have been several successful reports on tip-enhanced photoluminescence/fluorescence measurements using the contact-mode AFM^34–36^, some researchers have also reported that it is better to have a few nanometers of the tip-sample separation for strong signal enhancement in photoluminescence or fluorescence^37–40^. Therefore, we expect that the small-amplitude tapping-mode AFM that we proposed would be an effective way not only for Raman measurements but also for photoluminescence or fluorescence measurements.

In conclusion, we quantitatively investigated the influence of cantilever tapping amplitude on TERS signal in the amplitude-controlled tapping mode. The tapping amplitude significantly affected the TERS signal intensity. We found through numerical investigations that in the case of a tapping amplitude of 50 nm, which is a typical value used in a tapping-mode AFM, TERS intensity is reduced by as much as 80% compared with the contact mode. It was significantly improved to a level almost comparable to that of the contact mode by reducing the amplitude to a few nanometers. In addition, we experimentally verified that amplitude control is essential for the tapping-mode TERS measurements. TERS signal intensities were three times different between the tapping amplitudes of 1.6 nm and 24 nm. Discussions were also made to further advance the tapping-mode TERS techniques by considering the conditions of the tapping-mode AFM operation. The tapping mode is highly beneficial for investigating soft and fragile samples using TERS, although the contact mode has been the standard mode of AFM operation for TERS over the past few decades. This fundamental but practical investigation would highly stimulate diverse research fields for the further development of TERS as well as related nanophotonics techniques.

## Methods

### TERS measurement

A single-mode continuous-wave laser (wavelength: 638 nm, DL640-050-SO, CrystaLaser) was used for TERS measurements. It passes through a beam expander and several filters, such as ND filters, wave plates, and a z-polarizer. A spatial mask was also inserted for evanescent illumination of the metallic tip, which effectively suppressed the scattering noise from the metallic tip shaft. After being reflected by an edge filter and Galvano mirror scanner (6220H, Cambridge Technology), it was focused on the metallic tip through an oil-immersion objective lens (NA: 1.45, × 100, Olympus). The tip was mounted on an atomic force microscope (MFP-3D-BIO, Oxford Instruments) on an inverted optical microscope (ECLIPSE Ti2, Nikon). The position of the laser focus was precisely adjusted to the tip apex using a Galvano mirror scanner and relay optics to excite near-field light at the tip apex. Raman signal excited by the near-field light was detected by a CCD camera (PIXIS100B_eXcelon, Teledyne) through a spectroscope (HRS-300, Teledyne). Strong Rayleigh-scattered noise was efficiently removed by the edge filter. For the TERS measurement, the laser power and exposure time were ~ 1.0 mW at the sample plane and 5 s, respectively. The tapping amplitude was controlled by the voltage applied to the oscillation piezo.

### Tip fabrication

The metallic tips were fabricated by physical vapor deposition (VPC-1100, ULVAC) of silver on commercially available tapping-mode cantilever tips. The deposition rate was 0.05 nm/s, and the thickness was 60 nm. Silver nanoparticles were formed on the tip under these conditions, which facilitated the generation of strong near-field light through localized plasmon resonance. Three types of silicon cantilever tips of different lengths were used in this study. The first was a cantilever with a length of 160 µm (OMCL-AC160TN, Olympus, cantilever width: 40 µm, cantilever thickness: 3.7 µm, spring constant: 26 N/m, resonance frequency: 300 kHz). The other two cantilevers were a 240-µm-long cantilever (OMCL-AC240TN, Olympus, cantilever width: 40 µm, cantilever thickness: 2.3 µm, spring constant: 2 N/m, resonance frequency: 70 kHz), and a 55-µm-long cantilever (OMCL-AC55TN, Olympus, cantilever width: 31 µm, cantilever thickness: 2.35 µm, spring constant: 85 N/m, resonance frequency: 1,600 kHz).

### Preparation of a few-layered WS_2_

The cover slips were cleaned with piranha solution for 30 min at 120 °C. First, a copper buffer layer with a thickness of 2 nm was deposited on the cleaned cover slip via physical vapor deposition (VPC-1100, ULVAC). Subsequently, gold was evaporated to a thickness of 10 nm. The copper layer acts as an adhesion layer to form a smooth gold layer. The evaporation rates were 0.5 nm/s for both metals. Few-layered WS_2_ was prepared from a WS_2_ bulk crystal (BLK-WS2, 2D Semiconductors) by mechanical exfoliation using scotch tape. Once the few-layered WS_2_ samples were prepared on the scotch tape, they were transferred to a thermal-release tape. We pressed the thermal-release tape on a gold-coated cover slip using a tweezer. Only the thermal-release tape was removed by heating it to 100 °C on a hot plate to avoid damaging and contaminating the WS_2_ samples or the gold layer.

## Data Availability

The datasets used and/or analysed during the current study available from the corresponding author (T.U.) on reasonable request.
